# Effects of pH and Osmotic Changes on the Metabolic Expressions of *Bacillus subtilis* Strain 168 in Metabolite Pathways including Leucine Metabolism

**DOI:** 10.3390/metabo12020112

**Published:** 2022-01-25

**Authors:** Min-Kyung Park, Soyeon Lee, Young-Suk Kim

**Affiliations:** Department of Food Science and Biotechnology, Ewha Womans University, Seoul 03760, Korea; carrot0412@gmail.com (M.-K.P.); lunaleefs@gmail.com (S.L.)

**Keywords:** *Bacillus subtilis*, pH, osmosis, leucine, metabolic changes

## Abstract

*Bacillus subtilis* is often exposed to diverse culture conditions with the aim of improving hygiene or food quality. This can lead to changes in the volatile metabolite profiles related to the quality of fermented foods. To comprehensively interpret the associated metabolic expressions, changes in intracellular primary and extracellular secondary volatile metabolites were investigated by exposing *B. subtilis* to an alkaline pH (BP, pH 8.0) and a high salt concentration (BS, 1 M). In particular, *B. subtilis* was cultured in a leucine-enriched medium to investigate the formation of leucine-derived volatile metabolites. This study observed metabolic changes in several metabolic pathways, including carbohydrate metabolism, amino acid metabolism, fatty acid metabolism, and leucine degradation. The formation of proline (an osmolyte), furans, pyrrole, and monosaccharide sugars (glucose, galactose, and fructose) was enhanced in BS, whereas fatty acid derivatives (ketones and alcohols) increased in BP. In the case of leucine degradation, 3-methyl-butanal and 3-methylbutanol could be salt-specific metabolites, while the contents of 3-methylbutanoic acid and 3-methylbutylacetate increased in BP. These results show culture condition-specific metabolic changes, especially secondary volatile metabolites related to the sensory property of foods, in *B. subtilis*.

## 1. Introduction

Various fermented foods are consumed worldwide due to their health benefits and flavor. During fermentation, raw food ingredients are converted into useful edible materials by breaking down macromolecules, such as carbohydrates, proteins, and lipids, into micromolecules, which can be precursors to the volatile metabolites related to food flavor. The main metabolites that determine fermented food quality are mostly produced via metabolic reactions involving the various enzymes in fermentative microorganisms. Each microorganism has a specific cellular metabolic system, such as a catabolic or anabolic system. The secretion of a metabolite to the extracellular medium can be induced by the overflow of intracellular metabolites. Since intracellular metabolites represent the reactants of a metabolic network, knowledge of changes therein can provide a comprehensive understanding of extracellular metabolite production [1]. Thus, a metabolomic approach, which quantifies internal and external metabolites, is important for explaining the metabolic responses to specific variables [2].

*Bacillus subtilis* is a Gram-positive endospore-forming bacterium that is widely used for industrial purposes, such as the production of enzymes, antibiotics, and fermented foods [3]. In particular, studies of *B. subtilis* have strongly focused on fermented food flavors, such as those of fermented soybeans, for example, *dawadawa* [4], chunggukjang [5], and natto [6]. However, there have been few studies on the related metabolic changes, especially those relating to volatile metabolites formation after exposure to various environmental changes, despite *B. subtilis* being frequently exposed to high osmolarities or changes in pH levels to improve the efficiency of the process and hygiene. Exposing *B. subtilis* to a high salt concentration and alkaline pH has been widely applied for industrial purposes to promote enzyme activity or control its growth. *B. subtilis* can adapt efficiently to various stresses, such as those from pH and osmosis [7]. The optimum pH of its protease is between 8 and 10 [8], whereas osmotic stress can affect its growth conditions and enzyme activity. It is therefore important to understand the metabolomic responses of *B. subtilis* to different culture conditions.

This study investigated the effects of culture conditions, such as alkaline pH (BP) and high salinity (BS), on the metabolome changes of *B. subtilis* strain 168. Kohlstedt et al. [9] previously studied the adaption of *B. subtilis* to osmotic challenge using multi-omics, such as transcriptome, proteome, metabolome, and fluxome. The changes in carbon core metabolism were elucidated, indicating the altered metabolism for the formation of proline, a precursor of glutamate. In general, microorganisms grow optimally within a specific pH range and depend on the pH for enzymatic activities [10]. Accordingly, the formation of metabolites in *B. subtilis* can be varied depending on pH. However, there is a lack of studies regarding metabolomic alterations depending on cultivation conditions such as pH and high salt, focusing on amino acids metabolism, in particular, related to secondary volatile metabolites in *B. subtilis*, although some studies have investigated the effects of pH and osmotic changes on the metabolome as well as genome. In this study, *B. subtilis* was cultivated in a leucine-enriched medium, since leucine contributes to the formation of diverse volatile metabolites, such as fusel aldehydes, alcohols, acids, esters, and nitrogen-containing compounds [11]. Leucine metabolism has also been studied since leucine plays diverse roles in providing essential substrates for new proteins and is a key regulator of functional reactants, such as muscle and insulin synthesis [12,13].

In this study, *B. subtilis* strain 168 was used as a metabolic model and it has its own database, including biochemical reactions, regulatory networks, and metabolic pathways [14]. A metabolomics approach (the quantification of intracellular primary and extracellular secondary volatile metabolites) was used to investigate the molecular adaptation to changes in culture conditions. This approach could provide insight into the specific metabolic responses of *B. subtilis* according to the culture conditions. In particular, the changes of secondary volatile metabolites provide fundamental information for predicting the effects of fermentation on food quality, because volatile secondary can be highly related to the sensory property of fermented foods. This study aimed to study the culture condition-specific metabolic changes in *B. subtilis* by investigating the quantitative changes of intracellular primary metabolites and extracellular secondary volatile metabolites. Intracellular metabolites reflect the flux of metabolic networks, whereas extracellular metabolites show the accumulated reactants of them.

## 2. Results

The changes of primary metabolites and secondary volatile metabolites depend on culture conditions, such as alkaline pH (pH 8.0) and high salt concentration (1 M NaCl), in *B. subtilis.* The growth of *B. subtilis*, cultivated using different culture conditions, was observed according to culture time (Figure S1). The sampling points were set as the exponential phase (Phase Ⅰ), early stationary phase (Phase Ⅱ), and later stationary phase (Phase Ⅲ). During cultivation, BC (control) and BS (1 M salt concentration) showed similar growth patterns, while the time required for the stationary phase of BP (pH 8.0) was shorter than those for the other samples, which indicated that pH levels affected the growth of *B. subtilis* more significantly compared to salt concentration. In this study, cell pellets were harvested for intracellular primary metabolites, while a culture medium separated from the cells was used for extracellular secondary volatile metabolite analysis [1].

### 2.1. Analysis of Primary and Secondary Volatile Metabolite Profiles in B. subtilis

GC–TOF/MS was used to identify 34 primary metabolites, including 7 sugars, 19 amino acids and derivatives, 2 fatty acids, and 6 organic acids, produced by *B. subtilis*. The identification of secondary volatile metabolites in *B. subtilis* was conducted using GC–MS. A total of 61 secondary volatile metabolites, including 12 acids, 10 alcohols, 9 esters, 14 ketones, 3 aldehydes, 4 furans, 5 nitrogen-containing compounds, and 4 benzenes, were obtained. This study used principal component analysis (PCA) and orthogonal projections to latent structures-discriminant analysis (OPLS-DA) to investigate the differences in the metabolic responses of *B. subtilis* associated with changes in culture conditions. PCA (Figure S1) reveals sample group distributions based on each metabolic feature of primary metabolites and secondary volatile metabolites, and provides an overview of the changes in primary metabolites and secondary volatile metabolites based on culture conditions. Metabolic change patterns according to each phase were observed in the PCA results. PCA is commonly used as a preprocessing step in order to obtain a compact representation of a dataset. The direction of the first and second components in PCA explains the most variation of datasets. [15]. Figure S2A indicates that all samples had a trend of moving in the positive direction along the t [1] dimension according to culture times. The direction of each sample varied depending on the culture conditions shown in Figure S2B. This might explain why the basic metabolism, including carbohydrate (glycolysis and TCA cycle), amino acid, and fatty acid metabolism, is similar in identical microorganisms. However, metabolic profiles, especially secondary volatile metabolites, were highly affected by culture conditions.

**Figure 1:**
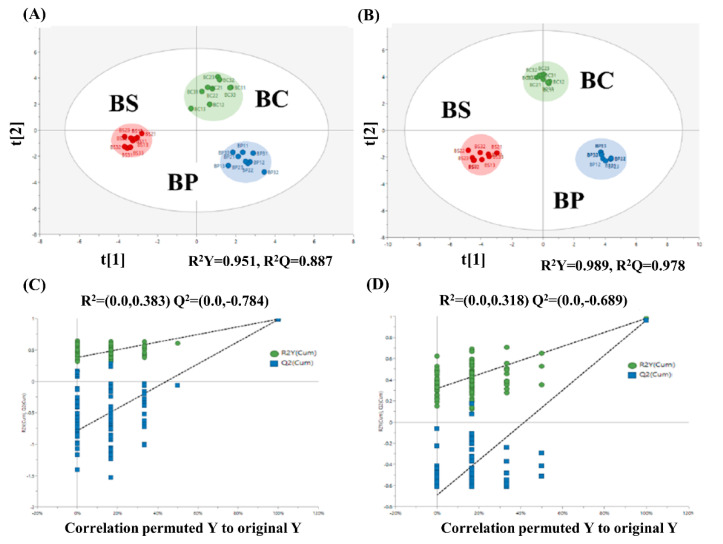
OPLS-DA score scatter plot of control (BC), alkaline pH (BP), and high salt concentration (BS) groups based on primary metabolite profiles (**A**) and secondary volatile metabolite profiles (**B**) obtained according to culture times. Permutation test plots based on primary metabolites (**C**) and secondary volatile metabolites (**D**) were conducted. Colors represent the following: red-BS, blue-BP, and green-BC. The meaning of the letters and numbers inside the score plot are the following: BC11-13, 21-23, and 31-33 (control samples obtained at phases Ⅰ, Ⅱ, and Ⅲ in replication, respectively), BP11-13, 21-23, and 31-33 (BP samples obtained at phases Ⅰ, Ⅱ, and Ⅲ in replication, respectively), and BS11-13, 21-23, and 31-33 (BS samples obtained at phases Ⅰ, Ⅱ, and Ⅲ in replication, respectively).

OPLS-DA models (Figure 1) were used to explain the statistically significant differences of primary metabolic profiles (A) and secondary volatile metabolic profiles (B) in each group depending on the culture conditions. The OPLS-DA model is useful for identifying significantly different metabolites, since it can maximize the distinction between samples to extend the regression of the PCA [16]. The OPLS-DA models demonstrated proper modeling and predictive abilities using one predictive component and two orthogonal components (A: R^2^Y = 0.951, Q^2^ = 0.887; B: R^2^Y = 0.989, Q^2^ = 0.978). Figure 1C,D show the permutation test plots of OPLS-DA models. The Q^2^ values (Q^2^ < 0) indicate that this model has no modeling error, such as overfitting, and the general requirement of a good model is R^2^X > 0.4 [17]. All of the Q^2^ and R^2^X values in this study were suitable for the requirements of OPLS-DA models.

**Figure 2:**
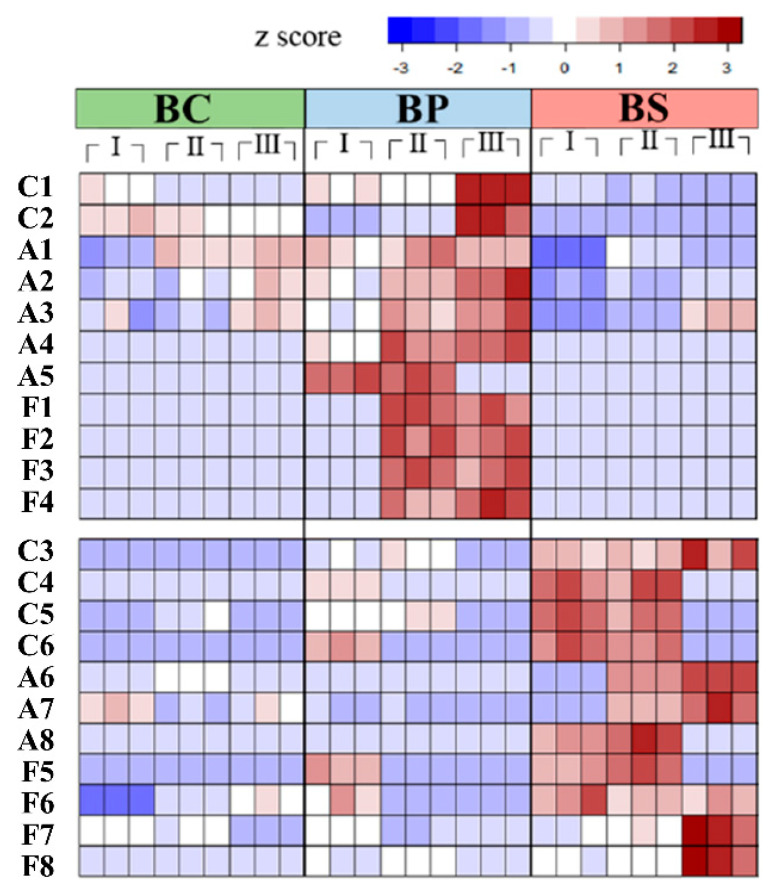
The heatmap shows the z-score value of each metabolite selected by VIP score and pcorr values (VIP > 1 and ΙpcorrΙ > 0.5) of the OPLS-DA model, and color intensity represents the metabolic expressions: red represents the up-expression and blue the down-expression. Main metabolism was divided into three parts: C: carbohydrate metabolism, A: amino acid metabolism, and F: fatty acid metabolism. Abbreviations are as follows: BC: control sample, BP: cultivation under alkaline pH (pH 8.0), BS: cultivation under high salt concentration (1 M salt concentration), Ⅰ: exponential time (phase Ⅰ), Ⅱ: early stationary time (phase Ⅱ), and Ⅲ: later stationary time (phase Ⅲ). Boxes in each phase (Ⅰ, Ⅱ, and Ⅲ) represent replicas of the measurements.

The OPLS-DA results indicate that different sample groups were located in different dimensions. The scoring of the second latent variables indicated that BC was independent of BP and BS in both models, and the first latent variables indicated a clear separation between BS (salt) and BP (pH level). The projection (VIP) values and correlation coefficient value (pcorr), of variable importance, were used to select 22 variants that would determine the metabolic characteristics of each sample (VIP > 1 and ΙpcorrΙ > 0.5). To investigate the influences of culture conditions, the most important indicator variables from the OPLS-DA model were listed and categorized by their most relevant metabolic system, such as carbohydrate, amino acid, and fatty acid metabolism (Figure 2 and Table S1).

In Figure 2, the heatmap was visualized by the z-score of metabolite formation. The abundance of each metabolite was indicated using red (up-expressed) and blue (down-expressed). The color shade changed based on culture conditions and growth phases. The amount of each metabolite generally increased with the culture time, although some metabolites decreased at later stationary phases. This might be explained by the large accumulation of metabolites converting into other metabolites or being affected by different metabolic systems.

The formation of sugars, such as fructose and galactose, increased more significantly with culture times in BP than the other samples. 3-Methylbutyl acetate was only found in BP during phases Ⅰ and Ⅱ. 3-Methylbutylacetate is derived from a branched chain amino acid (leucine) via the action of alcoholacetyltransferases [18]. This enzyme can convert branched alcohols into branched acetates. However, 3-methylbutylacetate was not found at phase Ⅲ of BP. It can be assumed that pH levels and the accumulation of metabolites influence the formation of 3-methylbutylacetate in *B.subtilis*. Osmolyte accumulation in BS is important since it increases salt stress tolerance in bacteria cells. Many bacteria synthesize and accumulate some osmolytes, such as proline [19], glycine-betaine [20], and trehalose [21]. In particular, proline is considered the most widely distributed osmolyte accumulated under salt stress in *B. subtilis* [22]. In the present study, the contents of proline and ornithine (precursor of proline synthesis) increased more in BS compared to the other samples.

### 2.2. The Changes of Metabolic Pathways in Bacillus subtilis

**Figure 3:**
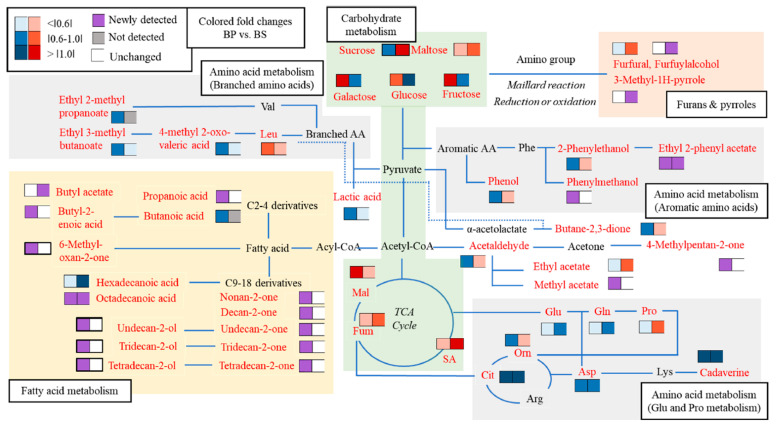
The possible metabolic pathways of specific metabolites obtained at phase Ⅲ in *B. subtilis*. The fold changes (>1.5) compared to BC were visualized by heatmaps. Metabolites showing significant changes compared to the control are written in red letters. Up-expressed metabolites are represented by a red color, while down-expressed metabolites are shown in a blue color. Abbreviations are as follows: BP: alkaline pH; BS: high salt concentration; Glu: glutamic acid; Gln: glutamine; Pro: proline; Asp: aspartic acid; Orn: ornithine; Lys: lysine; Fum: fumaric acid; Mal: malic acid; SA: succinic acid; Val: valine, Phe: phenylalanine; Leu: leucine.

Accumulated metabolites can be converted into other metabolites or affect other metabolic systems due to their complexity. Figure 3 and Table S3 shows the significant changes (Ιfold changeΙ > 1.5) in the formation of primary metabolites and secondary volatile metabolites compared with BC according to their possible metabolic pathways, as proposed in previous studies [23,24,25]. Although the isolation of related enzymes was excluded from this study, the accumulated changes of metabolites could explain the pattern of metabolic flux.

The metabolites obtained at phase Ⅲ were compared as the metabolic expression results of each phase were shown cumulatively. The intensities of the log_2_-transformation were normalized to colored fold changes in each metabolite. The red and blue colors represent up-expressed and down-expressed metabolites, respectively. The purple and white colors represent newly detected and unchanged metabolites compared with BC. The gray color represents metabolites that were not detected. Metabolic pathways can be separated into three parts: carbohydrate (light green: glycolysis and TCA cycle), amino acid (gray: aromatic amino acid, glutamic acid and proline, and branched amino acid metabolism), and fatty acid (orange: short- and long-chain fatty acid and derivatives) metabolic systems.

Carbohydrate metabolism, such as glycolysis and TCA cycle, provides the precursors for various primary and secondary volatile metabolites. During cultivation, alkaline pH and salt concentration may have caused increases in sugars synthesis or decreases in carbohydrate degradation in *B. subtilis*. The contents of sucrose (carbon source) decreased more significantly in BP than in BC. Meanwhile, sugars, such as galactose, glucose, and fructose, increased in BP, whereas BS showed a decrease in monosaccharide sugars compared with BC. In addition, organic acids which are components of the TCA cycle (malic acid, succinic acid, and fumaric acid), increased more considerably in BP and BS than those in BC.

Compared with BC, amino acid metabolisms varied in both samples. In glutamic acid and proline metabolism, proline increased, while glutamate and glutamine decreased, and ornithine slightly increased in BS. In branched amino acid metabolism, the content of leucine increased, whereas those of branched amino acid derivatives, including 4-methyl-2-oxo valeric acid (KICA), ethyl 2-methyl propanoate, and ethyl 3-methyl butanoate, decreased in BP. 2-Phenylethanol, a rose-scented volatile metabolite, was down-expressed, while phenylmethanol and ethyl 2-phenyl acetate were up-expressed in BP.

Many fatty acid-derived volatile metabolites could be attributed to carbohydrate metabolism and enzymatic activities (β-oxidation, decarboxylation, and other oxidation/reduction). For example, fatty ketones (nonan-2-one, decan-2-one, undecane- 2-one, tridecan-2-one, and tetradecane-2-one) and alcohols (undecane-2-ol, tridecan-2-ol, and tetradecane-2-ol) are synthesized from the beginning of fatty acid biosynthesis via the carboxylation pathway of acetyl-CoA to malonyl-CoA and acyl-CoA [26]. After fatty acid biosynthesis, the carboxylic group of fatty acids are converted into alcohol or ketone by reductase [27]. Ketones and alcohols derived from long-chain fatty acids were first detected in BP.

### 2.3. Leucine Metabolism

**Figure 4:**
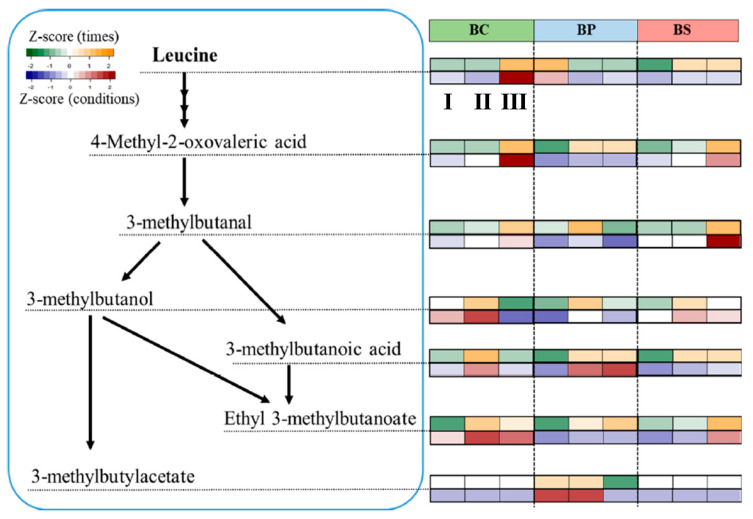
Leucine degradation pathway, reflecting the metabolite changes obtained at phases Ⅰ–Ⅲ in *B. subtilis* under different cultivation conditions (BC: control; BP: alkaline pH; BS: high salt concentration). The colors (green and orange) represent the changes of metabolites according to culture times in each condition. Blue and red colors represent the metabolic response to culture conditions.

Figure 4 depicts the changes in leucine-derived secondary volatile metabolites, such as 4-methyl-2-oxovaleric acid (KICA), 3-methylbutanal, 3-methylbutanoic acid, ethyl 3-methylbutanoate, and 3-methylbutylacetate, according to culture times and conditions. The heatmap indicates the correlation between metabolic expressions and culture times or conditions. The color intensity represents the z-score range of metabolic expressions: the red and orange colors indicate up-expression, and blue and green indicate down- expression (Table S3).

In BC, the contents of leucine (a precursor), KICA (an intermediate), 3-methylbutanal (a product), and ethyl 3-methylbutanoate (a product) increased with the culture time, while 3-methylbutanoic acid and ethyl 3-methylbutanoate were up-expressed in BP during cultivation. 3-Methylbutyl acetate (isoamyl acetate), which has a strong banana- and pear-like odor [28], was only detected in BP; however, it was not detected at phase Ⅲ. The formation of 3-methylbutanal, 3-methylbutanoic acid, and ethyl 3-methyl- butanoate increased in BS with the culture time. BS showed similar leucine degradation patterns to BC, except for 3-methylbutanol.

Among secondary volatile metabolites, 3-methylbutanal, 3-methylbutanoic acid, and 3-methylbutylacetate were strongly related with pH levels or high salt concentration. 3-Methylbutanal was greatly up-expressed in BS comparing with BC and BP. It was converted from KICA via the decarboxylation reaction [29], and it can be an intermediate in the synthesis pathways of 3-methylbutanol and 3-methylbutanoic acid. The metabolic expression of 3-methylbutanol, which can be induced from 3-methyl-butanal by alcohol dehydrogenase, was slightly enhanced in BS during cultivation. Meanwhile, 3-methtylbutanoic acid was significantly up-expressed in BP compared with the other samples. These results indicated that the formation of secondary volatile metabolites was noticeably changed according to culture conditions.

## 3. Discussion

This study has revealed the effects of culture conditions of alkaline pH and high salt concentration on the formation of primary metabolites and secondary volatile metabolites in *B. subtilis*. Specific conditions (pH 8.0 and 1 M salt concentration) were selected following the preliminary test. At pH 8.0, the activity of protease was considerably promoted compared to control, whereas it decreased at high salt concentration. We selected the experimental conditions with different protease activity, which would significantly affect amino acid metabolism.

The quantification of intracellular and extracellular metabolites is important for understanding cellular metabolism, which reveals the correlation between stimulus and metabolic responses. Metabolic pathways encompass a series of biochemical reactions and are prerequisites to their final or intermediate products. For example, carbohydrate metabolism is connected with fatty acid and amino acid (branched amino acid and aromatic amino acid degradation) synthesis. Pyruvate can be converted into α-acetolactate and diacetyl, and also to acetyl-coA, which can be converted into fatty acids and their derivatives. α-Acetolactate can be converted to a branched amino acid (leucine) or diacetyl. This study focused on significant metabolic changes based on the possible metabolic pathways of *B. subtilis*.

Ketones and alcohols, derived from fatty acids and carbohydrate derivatives (sugars and TCA cycle metabolites), increased in BP, while the contents of proline, furans, and pyrrole increased in BS. These results clearly demonstrate that certain types of stress could induce different effects on metabolite formation during cultivation. In addition, monosaccharides (galactose, glucose, and fructose) and organic acids, which are components of the TCA cycle (malic acid, succinic acid, and fumaric acid), increased more considerably in BP than in BC, whereas the contents of sucrose decreased. Sucrose can be hydrolyzed to glucose and fructose by invertases. Zhou et al. (2016) [30] reported novel alkaline invertases which were isolated from *Bacillus* sp. Thus, it might be considered that alkaline invertases of *B. subtilis* affect the increase in monosaccharides (glucose and fructose) and the decrease in sucrose in BP.

Some metabolites, such as furfural, furfuryl alcohol, and 3-methyl-1H-pyrrole, which are generally related to sugar and/or amino acid degradation, which is increased in BS. In particular, furfural can be derived from lignocellulosic biomass and can be converted into furfuryl alcohol, 2-furoic acid, levulinic acid, or furans via the reduction or oxidation of the carbonyl group [31]. Pyrroles are synthesized via the cyclization of 1,4-dicarbonyl compounds with an excess of ammonia or primary amines [32]. It might be related to amino acid degradation in the biosystem, producing ammonia or primary amines.

Proline is the only compatible solute that *B. subtilis* can synthesize de novo in response to osmotic stress [33]. Ornithine, glutamate and proline are inter-convertible amino acids, and the direction of their formations could depend on metabolic requirements and supply of nitrogen [34]. In this study, the contents of proline and ornithine which is precursor to proline synthesis increased in BS during cultivation. However, glutamate, which is an inter-convertible amino acid, decreased. This demonstrates that the proline metabolism of *B. subtilis* is greatly affected by the salt concentration. The accumulation of proline has been generally correlated with tolerance to stresses [35]. On the contrary, the content of proline decreased in BP in this study. However, the possible explanation on its reduction remains unclear.

In this study, leucine was a major nitrogen source to investigate the changes in the generation of leucine derivatives depending on the culture conditions during *B. subtilis* cultivation. Leucine metabolism consists of multiple biochemical reactions. Leucine is either synthesized in the intracellular system or provided by transport systems. Certain intermediates, such as KICA and isovaleryl-CoA, are then formed, and converted into important precursors for various secondary volatile metabolites [36]. KICA is especially involved in the pathway of the formation of secondary volatile metabolites, such as 3-methylbutanal, 3-methylbutanol, 3-methylbutanoic acid, and ethyl 3-methylbutanoate. Changes in leucine-derived secondary volatile metabolites based on culture conditions were investigated due to their possible contribution to the quality of fermented foods. For example, 3-methylbutanal has a very low odor threshold (malty-like) [29], and can be converted into alcohol and carboxylic acid by alcohol dehydrogenase and aldehyde dehydrogenase, respectively. 3-Methylbutanol has a whisky-like odor, while 3-methylbutanoic acid has an unpleasant odor (sweaty and rancid) [29]. 3-Methylbutanoic acid is derived from 3-methylbutanal by aldehyde dehydrogenase or other pathways, such as by converting α-hydroxy-isocaproate or isovaleryl-CoA [29]. Esterase induces the esterification of 3-methylbutan-1-ol to isoamyl acetate, and to ethyl 3-methylbutanonate [37]. In general, esters, which have fruity odors, can be converted into fusel alcohol or carboxylic acid [28]. The formation of leucine degradation products, such as 3-methylbutanal, 3-methylbutanaol, 3-methylbutanoic acid, ethyl 3-methylbutanoate, and 3-methylbutylacetate, varied according to the culture conditions. 3-Methylbutanoic acid and 3-methylbutyl acetate were characterized by BP, while 3-methylbutanal and 3- methylbutanol were strongly correlated with BS. In particular, 3-methylbutanoic acid, which is considered an off-odorant in many fermented foods, was a BP-specific metabolite in this study. pH control might therefore be an important factor to consider when attempting to improve the quality of flavors during *B. subtilis* fermentation.

In this study, the changes in culture conditions affected the flow of metabolic pathways. In particular, metabolic pathways related to monosaccharides and fatty acids showed significant differences in BP compared to BC. As we explained earlier, carbohydrate metabolism is connected to fatty acid metabolism. In our results, fatty acid-derived volatiles were enhanced in BP through the reduction of the carboxyl group of fatty acids to carbonyls, such as ketones. On the other hand, some acetates, such as ethyl 2-phenylacetate and butyl acetate, were highly expressed in BS. In leucine degradation, under alkaline stress, 3-methylbutanoic acid, which is considered a strong off-odorant, increased, while 3-methylbutanal, which is Strecker aldehyde of leucine, enhanced by osmotic stress. The difference in composition of volatile metabolites produced can significantly affect the quality of foods by fermentation. For instance, an increase in 3-methylbutanoic acid can lead to a negative perception of fermented foods, whereas an increase in ethyl 2-phenylacetate, which has rosy and honey-like odor notes [38], can contribute positively to the quality of some foods. This study demonstrated that *B. subtilis* fermentation could be considerably affected by specific cultivation conditions, leading to the change in quality of its fermented foods.

## 4. Materials and Methods

### 4.1. Chemicals and Reagents

Sucrose, potassium chloride, sodium nitrate, magnesium sulfate heptahydrate, and potassium phosphate were obtained from Samchun Pure Chemicals Co. Ltd. (Pyeongtaek, Gyeonggi-do, Korea). L-Threitol was purchased from Tokyo Chemical Industry Co. Ltd. (Tokyo, Japan). Peptone, yeast extract and tryptone soya broth (TSB) were purchased from Becton Dickinson (Sparks, MD, USA). Acetonitrile and methanol were purchased from J.T.Baker (Phillipsburg, NJ, USA). All the other chemicals were obtained from Sigma-Aldrich (St. Louis, MO, USA).

The synthetic medium contained 1% leucine, 2%, sucrose, 0.5% peptone, 0.1% yeast extract, 0.1% potassium phosphate, 0.05% of potassium chloride and magnesium sulfate heptahydrate, trace element (5 g of citric acid, 1 g of ZnSO_4_·H_2_O, 5 g of Fe(NH_4_)_2_ (SO_4_)_2_·6H_2_O, 250 mg of CuSO_4_·5H_2_O, 50 mg of boric acid, 50 mg of MnSO_4_ and 50 mg of Na_2_MoO_4_·2H_2_O in distilled water), and vitamin solution (4 g of inositol, 200 mg of pantothenic acid, 200 mg of choline, 100 mg of thiamine, 75 mg of pyridoxine, 75 mg of nicotinamide, 30 mg of riboflavin, 5 mg of *p*-aminobenzoic acid, 5 mg of ascorbic acid, 5 mg of folic acid and 5 mg of biotin in 50% ethanol).

### 4.2. Strain and Cultivation of Bacillus subtilis

*Bacillus subtilis* strain 168 (KCTC 1326) was provided by Korean Collection for Type Culture (KCTC, Jeongeup, Jeollabuk-do, Korea). The preliminary tests were conducted to select the appropriate culture conditions (pH and salt concentration) for this experiment. The selection criteria depended on the following: the OD value and the activity of protease. Under these selected conditions, *B. subtilis* could survive against the external stress and its protease activity was significantly different compared to control.

*B. subtilis* was inoculated (initial OD_600_ = 0.1) in a synthetic medium and incubated at 35 °C for 42 hrs. The samples were attributed to the following states: (1) control (BC), incubation in control media (pH 6.0); (2) alkaline condition (BP), initial pH of the media was adjusted to 8.0 using a Tris-HCl solution; and (3) high salt concentration (BS), adding sodium chloride (Sigma-Aldrich) to reach at desired final concentration (1 M) at pH 6.0. Samples were harvested for metabolome study according to cultivation times, such as exponential time (phase Ⅰ, OD_final_ = $\frac{1}{2}$OD_max_), early stationary time (phase Ⅱ, OD_final_ = OD_max_, OD value can be constant), and later stationary time (phase Ⅲ, before decreasing OD value).

### 4.3. Extraction Non-Volatile Primary Metabolites and GC–TOF/MS Analysis

The extraction of non-volatile primary metabolites was modified from a previous method [39]. The lyophilized cell mass was dissolved in mixed solvent (acetonitrile: water = 1:1) with glass beads (diam. 1.0 mm, Sigma-Aldrich). Then, it was vortexed vigorously, prior to centrifugation at 7000× *g* for 5 min at 4 °C. After centrifugation, the supernatant was transferred into an Eppendorf tube with internal standards, such as L-threitol (for sugars and sugar alcohols), 3-hydroxy-2-phenylpropanoic acid (for organic acids), L-norleucine (for amino acids), and heptadecanoic acid (for fatty acids). Then, it was vacuum-dried in Centri-Vap (Labconco Co., Kansas City, MO, USA). The residue was derivatized with methoxyamine hydrochloride (20 mg/mL) in pyridine (Sigma-Aldrich), before being silylated with N, O-bis(trimethylsilyl)-trifluoroacetamide (BSTFA) with 1% trimethyl chlorosilane (TMCS).

An Agilent 6890N GC (Agilent Technologies) coupled with PEGASUS Ⅲ TOF/MS (Leco, St. Joseph, MI, USA) was applied. A DB-5MS column (30 m length, 0.25 mm internal diameter, 0.25 μm film thickness, J&B Scientific, Folsom, CA, USA) was equipped and helium flowed at constant flow rate of 0.8 mL/min. In total, 1 μL of the derivatized extract was injected in the split-less mode. Oven temperature was maintained at 80 °C for 5 min and increased to 180 °C at a rate of 10 °C/min per 5 min, and then it was raised to 240 °C at a rate of 8 °C/min and ramped to 290 °C at a rate of 10 °C/min and maintained for 10 min. Inlet and transfer line temperatures were 270 °C and 260 °C, respectively. The mass spectra data were collected in EI mode using 70 eV and obtained with mass scan range of *m*/*z* 35–400 at a rate of 20 spectra/s. Non-volatile metabolites were confirmed by comparing their mass spectral data and retention time to those of authentic standard compounds. The quantification of non-volatile metabolites was conducted by comparing their peak areas to those of internal standard compounds.

### 4.4. Extraction of Volatile Secondary Metabolites and GC–MS Analysis

Cultured samples were divided to cells and medium by centrifugation at 3000× *g* for 30 min at 4 °C. The supernatant (8 mL) and 2 μL of 4-methylpentan-2-ol (an internal standard compound, 100 mg/L in methanol) were transferred into 10 mL glass vial (Agilent Technologies, Santa Clara, CA, USA). A polydimethylsiloxane-coated stir bar (PDMS twister, 10 mm length, 1.0 mm film thickness, Gerstel GmbH, Mülheim and der Rühr, Germany) and ethylene glycol/silicone coated stir bar (EG/Silicon twister, 10 mm length, Gerstel GmbH) were applied for stir bar sorptive extraction (SBSE).

For gas chromatography–mass spectrometry (GC–MS) analysis, an HP7890B GC (Agilent Technologies) coupled with 5977A mass spectrometry (Agilent Technologies) was applied. Samples were injected in split-less mode and separated in a stabilwax^®^ column (30 m length, 0.25 mm internal diameter, 0.25 μm film thickness, Restek, Bellefonte, PA, USA) using helium as a carrier gas (0.8 mL/min). The oven program was started initially at 40 °C (5 min) 130 °C at a rate of 162 4 °C/min, and then ramped up to 230 °C (5 min) at a rate of 4 °C/min. The mass scan range was 35 to 350 *m*/*z* and transfer line temperature was 250 °C.

The identification of metabolites was positively confirmed by matching the mass spectral data and retention times to those of authentic standard compounds. Otherwise, the corrected spectra were compared with a mass spectral database (NIST08 and Wiley9n.1) and corresponding retention index (RI) values of NIST Chemistry Webbook. Quantitative data were obtained by comparing their peak areas to that of the internal standard compound.

### 4.5. Statistical Analysis

Principal component analysis (PCA) and orthogonal partial least square discriminant analysis (OPLS-DA) were conducted to distinguish the differences of the metabolites of *B. subtilis* according to cultivation conditions using SIMCA 16 (Umetrics, Umea, Sweden). Heat map visualization based on the z -score distribution was shown using a heatmap.2 function in the glpot package implemented in R environment (version 4.0.4).

## 5. Conclusions

This study investigated the effects of different culture conditions (alkaline pH and 1 M NaCl) on the formation of primary and secondary volatile metabolites of *B. subtilis*. The results indicate that culture conditions, such as alkaline pH and high salt concentration, have significant effects on the metabolic systems, including leucine metabolism. In particular, volatiles derived from fatty acids and proline metabolism were emphasized in BP and BS, respectively, compared to BC. Additionally, in leucine degradation, 3-methylbutanoic acid, which is considered as a strong off-odorant, was increased in BP, while 3-methylbutanol and 3-methylbutanal, which are known as the main contributors to fermented foods, were enhanced in BS. These results are valuable because the different metabolic expressions can affect the quality of fermented foods.

Although it is not a common phenomenon, the formation of leucine-derived secondary volatile metabolites was occasionally enhanced during the fermentation of foods. Additionally, for an example, soybean, which is the main ingredient used in food fermentation, contains leucine-enriched amino acids. Our results can be helpful for understanding the metabolic changes related to these compounds according to some cultivation conditions. It could be also applied for the control of fermented foods’ quality, because 3-methylbutanoic acid can be negatively related to this, whereas 3-methylbutanol and 3-methylbutanal can enhance the sensory properties of fermented foods.

## Data Availability

Data is contained within the article or Supplementary Materials.

## Supplementary Materials

The following are available online at https://www.mdpi.com/article/10.3390/metabo12020112/s1, Figure S1: PCA score scatter plot of control (BC), alkaline pH (BP), and high salt concentration (BS) groups based on primary metabolite profiles (A) and secondary volatile metabolite profiles (B); Figure S2: Growth curve of *B. subtilis* strain 168 cultivated under different culture conditions according to cultivation time (BC: control; BP: alkaline pH; BS: high salt concentration); Table S1: Main variables contributing to discrimination between primary and secondary volatile metabolic profiles of *B. subtilis* fermentations; Table S2: Metabolites contributing to significant metabolic changes in metabolic pathways of *B. subtilis*; Table S3: The changes of metabolites derived from leucine according to culture conditions, such as alkaline pH and temperature.
