# Supplementary material for: Effects of pH and Osmotic Changes on the Metabolic Expressions of Bacillus subtilis Strain 168 in Metabolite Pathways including Leucine Metabolism
Source: Metabolites. 2022 Jan 25;12(2):112. doi: 10.3390/metabo12020112 (PMC8880083; doi:10.3390/metabo12020112)
Supplement: Supplementary file 1 [file metabolites-12-00112-s001.zip › metabolites-1488752-supplementary.pdf]

*metabolites*

MDPI

*Supplementary Material*

# Metabolic changes of *Bacillus subtilis* strain 168 according to culture conditions in metabolite pathways including leucine metabolism

**Min Kyung Park, Soyeon Lee and Young-Suk Kim**

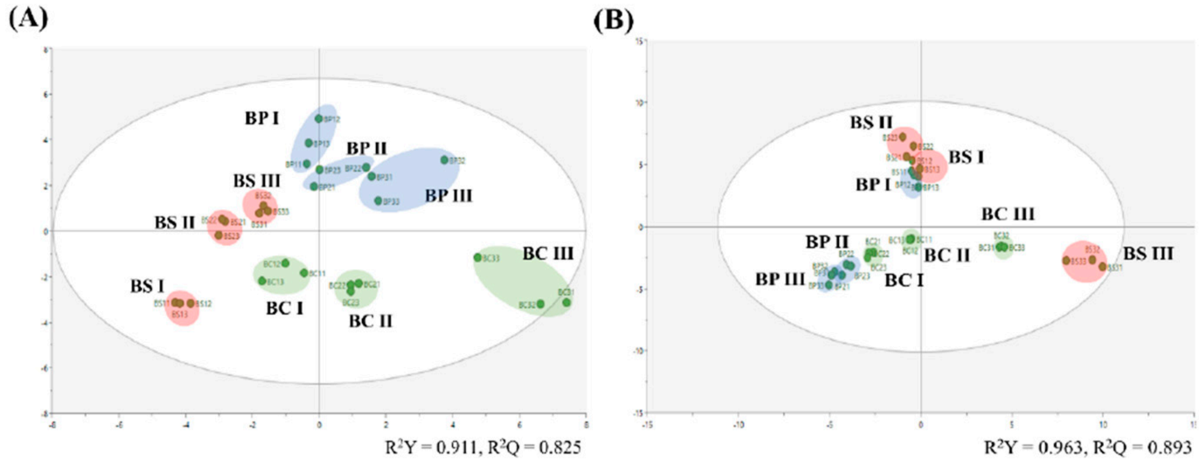


**Figure S1.** PCA score scatter plot of control (BC), alkaline pH (BP), and high salt concentration (BS) groups based on primary metabolite profiles (A) and secondary volatile metabolite profiles (B).

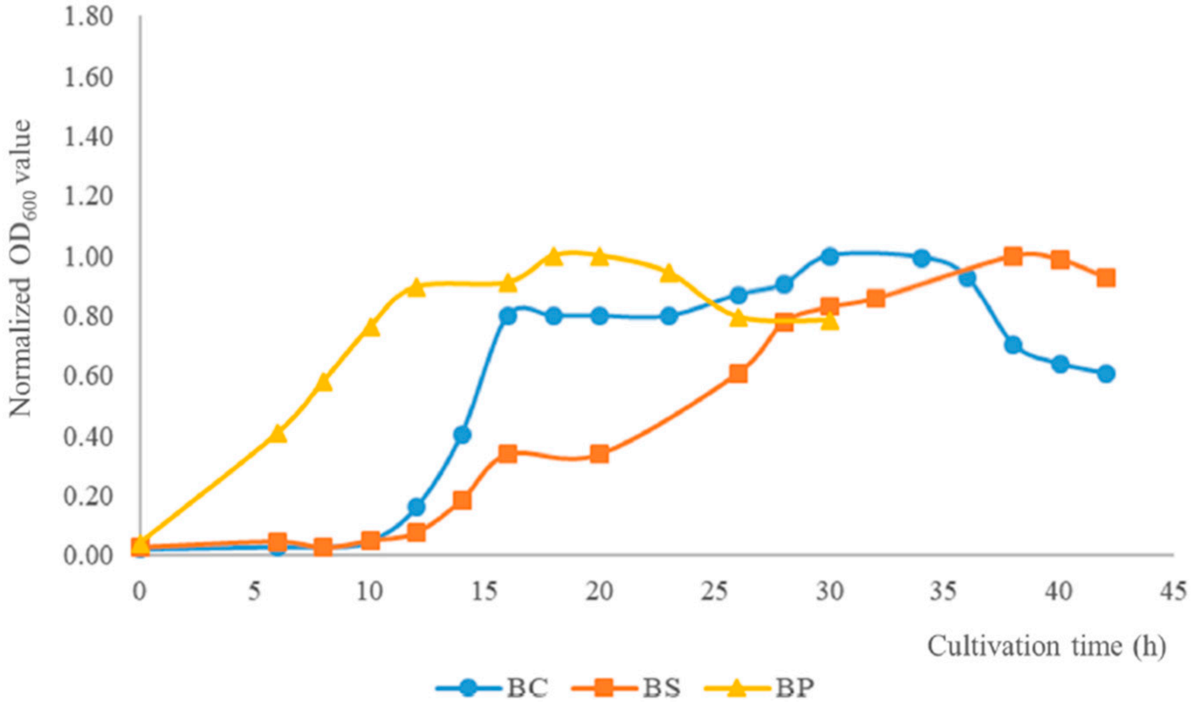

**Figure S2.** Growth curve of *B. subtilis* strain 168 cultivated under different culture conditions according to cultivation time (BC: control; BP: alkaline pH; BS: high salt concentration).

**Table S1.** Main variables contributing to discrimination between primary and secondary volatile metabolic profiles of *B. subtilis* fermentations.

| No | Metabolite | VIP | Z score values | | | | | | | | | Possible related metabolism |
|---|---|---|---|---|---|---|---|---|---|---|---|---|
| | | | BCⅠ | BCⅡ | BCⅢ | BPⅠ | BPⅡ | BPⅢ | BSⅠ | BSⅡ | BSⅢ | |
| | ***BP-specific*** | | | | | | | | | | | |
| C2 | Fructose | 1.14 | 0.14 | −0.36 | −0.62 | 0.26 | 0.03 | 2.58 | −0.6 | −0.66 | −0.76 | Carbohydrate metabolism |
| C4 | Galactose | 1.36 | 0.53 | 0.31 | −0.08 | −0.69 | −0.4 | 2.4 | −0.69 | −0.69 | −0.69 | Carbohydrate metabolism |
| A1 | Alanine | 1.07 | −0.89 | 0.57 | 0.71 | 0.36 | 1.29 | 0.92 | −1.65 | −0.31 | −1.01 | Amino acid metabolism |
| A4 | Isoleucine | 1.16 | −0.69 | −0.44 | 0.46 | 0.08 | 0.79 | 2.12 | −1.22 | −0.7 | −0.41 | Amino acid metabolism |
| A14 | Phenylalanine | 1.36 | −0.46 | −0.76 | 0.48 | −0.13 | 0.92 | 1.51 | −1.4 | −0.86 | 0.72 | Amino acid metabolism |
| v48 | Phenylmethanol | 1.28 | −0.6 | −0.6 | −0.6 | 0.13 | 1.56 | 1.93 | −0.6 | −0.6 | −0.6 | Amino acid metabolism |
| v12 | 3-Methylbutyl acetate | 1.16 | −0.52 | −0.52 | −0.52 | 1.88 | 1.78 | −0.52 | −0.52 | −0.52 | −0.52 | Amino acid metabolism |
| v33 | Undecan−2-one | 1.17 | −0.52 | −0.52 | −0.52 | −0.52 | 2 | 1.64 | −0.52 | −0.52 | −0.52 | Fatty acid metabolism |
| v43 | 6-Methyloxan-2-one | 1.16 | −0.52 | −0.52 | −0.52 | −0.52 | 1.88 | 1.74 | −0.52 | −0.52 | −0.52 | Fatty acid metabolism |
| v19 | Nonan−2-one | 1.15 | −0.51 | −0.51 | −0.51 | −0.51 | 1.94 | 1.66 | −0.51 | −0.51 | −0.51 | Fatty acid metabolism |
| v41 | Undecan-2-ol | 1.13 | −0.5 | −0.5 | −0.5 | −0.5 | 1.28 | 2.25 | −0.5 | −0.5 | −0.5 | Fatty acid metabolism |
| | ***BS-specific*** | | | | | | | | | | | |
| v37 | Furan-2-ylmethanol | 1.33 | −0.86 | −0.86 | −0.86 | −0.16 | 0.14 | −0.86 | 0.81 | 0.73 | 1.93 | Carbohydrate metabolism |
| v32 | Cyclopent-4-ene-1,3-dione | 1.13 | −0.62 | −0.62 | −0.62 | 0.33 | −0.62 | −0.62 | 1.66 | 1.72 | −0.62 | Carbohydrate metabolism |
| v56 | 3,5-Dihydroxy-6-methyl-2,3-dihydropyran-4-one | 1.09 | −0.79 | −0.3 | −0.79 | −0.13 | 0.22 | −0.79 | 1.88 | 1.48 | −0.79 | Carbohydrate metabolism |
| v55 | 4-Hydroxy-2,5-dimethylfuran-3-one | 1.11 | −0.67 | −0.67 | −0.67 | 0.95 | −0.67 | −0.67 | 1.75 | 1.34 | −0.67 | Carbohydrate metabolism |
| A5 | Proline | 1.16 | −0.62 | −0.02 | −0.35 | −0.62 | −0.64 | −0.57 | −0.68 | 1.27 | 2.22 | Amino acid metabolism |
| A15 | Ornithine | 1.07 | 0.61 | −0.63 | 0.12 | −0.76 | −0.6 | −0.74 | −0.97 | 0.92 | 2.05 | Amino acid metabolism |
| v35 | 1-Phenylethanone | 1.07 | −0.67 | −0.67 | −0.67 | 1.07 | −0.67 | −0.67 | 1.03 | 1.9 | −0.67 | Amino acid metabolism |
| v45 | 4-Methylpentanoicacid | 1.15 | −0.5 | −0.5 | −0.5 | −0.5 | −0.5 | −0.5 | 1.22 | 2.28 | −0.5 | Fatty acid metabolism |
| v26 | 2-Ethylhexan−1-ol | 1.11 | −1.59 | −0.31 | 0.23 | 0.56 | −0.9 | −0.93 | 1.45 | 0.64 | 0.85 | Fatty acid metabolism |
| v34 | Butanoic acid | 1.00 | 0.05 | −0.18 | −1.05 | 0.01 | −0.73 | −0.56 | −0.25 | 0.2 | 2.49 | Fatty acid metabolism |
| v11 | Butyl acetate | 1.08 | −0.6 | −0.6 | −0.6 | −0.2 | 0.12 | −0.6 | −0.19 | 0.08 | 2.6 | Fatty acid metabolism |
| v35 | 1-Phenylethanone | 1.07 | −0.67 | −0.67 | −0.67 | 1.07 | −0.67 | −0.67 | 1.03 | 1.9 | −0.67 | Amino acid metabolism |
| v45 | 4-Methylpentanoicacid | 1.15 | −0.5 | −0.5 | −0.5 | −0.5 | −0.5 | −0.5 | 1.22 | 2.28 | −0.5 | Fatty acid metabolism |
| v26 | 2-Ethylhexan-1-ol | 1.11 | −1.59 | −0.31 | 0.23 | 0.56 | −0.9 | −0.93 | 1.45 | 0.64 | 0.85 | Fatty acid metabolism |

| | | | | | | | | | | | | |
|---|---|---|---|---|---|---|---|---|---|---|---|---|
| v34 | Butanoic acid | 1.00 | 0.05 | −0.18 | −1.05 | 0.01 | −0.73 | −0.56 | −0.25 | 0.2 | 2.49 | Fatty acid metabolism |
| v11 | Butyl acetate | 1.08 | −0.6 | −0.6 | −0.6 | −0.2 | 0.12 | −0.6 | −0.19 | 0.08 | 2.6 | Fatty acid metabolism |

BC: control sample.

BP: cultivation under alkaline pH (pH 8).

BS: cultivation under high salt concentration (1 M salt concentration).

**Table S2.** Metabolites contributing to significant metabolic changes in metabolic pathways of *B. subtilis*.

| Metabolite | Relative contents (average) | | | Fold change | | Metabolite | Relative contents (average) | | | Fold change | |
|---|---|---|---|---|---|---|---|---|---|---|---|
| | BC | BP | BS | BP | BS | | BC | BP | BS | BP | BS |
| ***Primary metabolites*** | | | | | | ***Secondary volatile metabolites*** | | | | | |
| Leucine | 1.399 | 8.985 | 1.854 | 0.81 | 0.12 | Acetaldehyde | 14.466 | 1.682 | 24.483 | −0.93 | 0.23 |
| Proline | 1.873 | 0.819 | 14.299 | −0.36 | 0.88 | Propan-2-one | 2.48 | 0.614 | 2.913 | −0.61 | 0.07 |
| Methionine | 0.001 | 0.043 | 0.008 | 1.63 | 0.92 | Ethyl 3-methylbutanoate | 11.41 | 2.502 | 10.012 | −0.66 | −0.06 |
| Aspartic acid | 0.581 | 0.122 | 0.121 | −0.68 | −0.68 | Butyl acetate | NDa | ND | 5.79 | UCb | NeDc |
| Ornithine | 0.113 | 0.024 | 0.314 | −0.67 | 0.44 | Methyl acetate | ND | 0.318 | ND | NeD | UC |
| Glutamine | 5.402 | 2.855 | 0.695 | −0.28 | −0.89 | Nonan-2-one | ND | 1.061 | ND | NeD | UC |
| Glutamic acid | 5.405 | 2.853 | 0.685 | −0.28 | −0.9 | 2,3,5-Trimethylpyrazine | ND | ND | 0.163 | UC | NeD |
| Citrulline | 0.035 | 0.001 | 0.001 | −1.54 | −1.54 | Decan-2-one | ND | 0.567 | ND | NeD | UC |
| Cadaverine | 0.025 | 0.001 | 0.001 | −1.4 | −1.4 | 4-Methyl-2-oxovaleric acid | 2.391 | 0.288 | 1.478 | −0.92 | −0.21 |
| Lactic acid | 2.059 | 0.513 | 0.978 | −0.6 | −0.32 | Furan-2-carbaldehyde | 0.112 | 0.125 | 0.571 | 0.05 | 0.71 |
| Fumaric acid | 0.103 | 0.176 | 0.524 | 0.23 | 0.71 | 2,3,5,6-Tetramethyl-pyrazine | 0.751 | ND | ND | ND | ND |
| Phosphoric acid | 0.063 | 0.042 | 0.001 | −0.18 | −1.8 | 3-Methyl-1H-pyrrole | ND | ND | 0.738 | UC | NeD |
| Malic acid | 0.042 | 0.584 | 0.111 | 1.15 | 0.42 | Ethyl acetate | 1.491 | 1.132 | 6.48 | −0.12 | 0.64 |
| Succinic acid | 1.464 | 1.691 | 5.184 | 0.21 | 1.82 | Propanoic acid | ND | 0.429 | ND | NeD | UC |
| Fructose | 0.074 | 1.364 | 0.013 | 1.27 | −0.74 | Undecan-2-one | ND | 0.52 | ND | NeD | UC |
| Galactose | 0.046 | 0.694 | 0.007 | 1.18 | −0.84 | Butanoic acid | ND | 0.574 | 4.161 | NeD | NeD |
| Glucose | 0.013 | 0.067 | 0.001 | 0.7 | −1.12 | Dodecan-2-one | ND | 1.745 | ND | NeD | UC |
| Maltose | 0.019 | 0.053 | 0.119 | 0.45 | 0.81 | Tridecan-2-ol | ND | 1.053 | ND | NeD | NC |
| Sucrose | 0.053 | 0.019 | 0.119 | −1.48 | 1.17 | 4-Methylpentan-2-one | ND | 0.151 | ND | NeD | NC |
| ***Secondary volatile metabolites*** | | | | ***Secondary volatile metabolites*** | | | | | ***Secondary volatile metabolites*** | | |
| Furan-2-ylmethanol | ND | ND | 0.183 | NC | NeD | Tetradecan-2-ol | ND | 3.328 | Furan-2-ylmethanol | ND | ND |
| But-2-enoic acid | ND | 0.218 | ND | NeD | NC | 2-(2-hydroxyethoxy)-Ethanol | 3.507 | ND | But-2-enoic acid | ND | 0.218 |
| Undecan-2-ol | ND | 0.412 | ND | NeD | NC | Phenol | 2.326 | 0.405 | Undecan-2-ol | ND | 0.412 |
| Ethyl 2-phenylacetate | ND | 1.017 | 1.287 | NeD | NeD | (2E,6E)-3,7,11-Trimethyl-dodeca-2,6,10-trien-1-ol | ND | 1.081 | Ethyl 2-phenylacetate | ND | 1.017 |
| 6-Methyloxan-2-one | ND | 0.17 | ND | NeD | NC | Octadecanoic acid | ND | 4.81 | 6-Methyloxan-2-one | ND | 0.17 |
| Tridecan-2-one | ND | 1.041 | ND | NeD | NC | Tetradecanoic acid | 2.475 | ND | Tridecan-2-one | ND | 1.041 |

| | | | | | | | | | | | |
|---|---|---|---|---|---|---|---|---|---|---|---|
| Tetradecan-2-one | ND | 2.173 | ND | NeD | NC | Ethyl 2-methylpropanoate | 13.776 | 2.105 | Tetradecan-2-one | ND | 2.173 |
| Phenylmethanol | ND | 1.431 | ND | NeD | NC | Hexadecanoic acid | 25.485 | 14.58 | Phenylmethanol | ND | 1.431 |
| 2-Phenylethanol | 2.215 | 0.321 | 2.25 | −0.84 | 0.01 | Butane-2,3-dione | 23.422 | 2.455 | 2-Phenylethanol | 2.215 | 0.321 |
| | | | | | | 4-Methylpentan-2-one | ND | 0.151 | | | |
| ***Secondary volatile metabolites*** | | | | ***Secondary volatile metabolites*** | | | | | ***Secondary volatile metabolites*** | | |
| Furan-2-ylmethanol | ND | ND | 0.183 | NC | NeD | Tetradecan-2-ol | ND | 3.328 | Furan-2-ylmethanol | ND | ND |
| But-2-enoic acid | ND | 0.218 | ND | NeD | NC | 2-(2-hydroxyethoxy)-Ethanol | 3.507 | ND | But-2-enoic acid | ND | 0.218 |
| Undecan-2-ol | ND | 0.412 | ND | NeD | NC | Phenol | 2.326 | 0.405 | Undecan-2-ol | ND | 0.412 |
| Ethyl 2-phenylacetate | ND | 1.017 | 1.287 | NeD | NeD | (2E,6E)-3,7,11-Trimethyl-dodeca-2,6,10-trien-1-ol | ND | 1.081 | Ethyl 2-phenylacetate | ND | 1.017 |
| 6-Methyloxan-2-one | ND | 0.17 | ND | NeD | NC | Octadecanoic acid | ND | 4.81 | 6-Methyloxan-2-one | ND | 0.17 |
| Tridecan-2-one | ND | 1.041 | ND | NeD | NC | Tetradecanoic acid | 2.475 | ND | Tridecan-2-one | ND | 1.041 |
| Tetradecan-2-one | ND | 2.173 | ND | NeD | NC | Ethyl 2-methylpropanoate | 13.776 | 2.105 | Tetradecan-2-one | ND | 2.173 |
| Phenylmethanol | ND | 1.431 | ND | NeD | NC | Hexadecanoic acid | 25.485 | 14.58 | Phenylmethanol | ND | 1.431 |
| 2-Phenylethanol | 2.215 | 0.321 | 2.25 | −0.84 | 0.01 | Butane-2,3-dione | 23.422 | 2.455 | 2-Phenylethanol | 2.215 | 0.321 |
| | | | | | | 4-Methylpentan-2-one | ND | 0.151 | | | |
| ***Secondary volatile metabolites*** | | | | ***Secondary volatile metabolites*** | | | | | ***Secondary volatile metabolites*** | | |
| Furan-2-ylmethanol | ND | ND | 0.183 | NC | NeD | Tetradecan-2-ol | ND | 3.328 | Furan-2-ylmethanol | ND | ND |
| But-2-enoic acid | ND | 0.218 | ND | NeD | NC | 2-(2-hydroxyethoxy)-Ethanol | 3.507 | ND | But-2-enoic acid | ND | 0.218 |
| Undecan-2-ol | ND | 0.412 | ND | NeD | NC | Phenol | 2.326 | 0.405 | Undecan-2-ol | ND | 0.412 |
| Ethyl 2-phenylacetate | ND | 1.017 | 1.287 | NeD | NeD | (2E,6E)-3,7,11-Trimethyl-dodeca-2,6,10-trien-1-ol | ND | 1.081 | Ethyl 2-phenylacetate | ND | 1.017 |
| 6-Methyloxan-2-one | ND | 0.17 | ND | NeD | NC | Octadecanoic acid | ND | 4.81 | 6-Methyloxan-2-one | ND | 0.17 |
| Tridecan-2-one | ND | 1.041 | ND | NeD | NC | Tetradecanoic acid | 2.475 | ND | Tridecan-2-one | ND | 1.041 |
| Tetradecan-2-one | ND | 2.173 | ND | NeD | NC | Ethyl 2-methylpropanoate | 13.776 | 2.105 | Tetradecan-2-one | ND | 2.173 |

| | | | | | | | | | | |
|---|---|---|---|---|---|---|---|---|---|---|
| Phenylmethanol | ND | 1.431 | ND | NeD | NC | Hexadecanoic acid | 25.485 | 14.58 | Phenylm ethanol | ND | 1.431 |
| 2-Phenylethanol | 2.215 | 0.321 | 2.25 | −0.84 | 0.01 | Butane-2,3-dione | 23.422 | 2.455 | 2-Phenylet hanol | 2.215 | 0.321 |

(BC: control; BP: alkaline pH; BS: high salt concentration).

[a] Not detected.

[b] Unchanged metabolite compared with BC (control).

[c] Newly detected metabolite compared with BC.

**Table S3.** The changes of metabolites derived from leucine according to culture conditions, such as alkaline pH and temperature.

| Metabolite | Z-score | | | | | | | | |
|---|---|---|---|---|---|---|---|---|---|
| | **BC Ⅰ** | **BCⅡ** | **BCⅢ** | **BP Ⅰ** | **BPⅡ** | **BPⅢ** | **BS Ⅰ** | **BSⅡ** | **BSⅢ** |
| ***Time-dependent*** | | | | | | | | | |
| Leucine | −0.61 | −0.72 | 1.32 | 1.24 | −0.71 | −0.53 | −1.32 | 0.69 | 0.62 |
| Ketoisocaproate | −0.81 | −0.51 | 1.31 | −1.32 | 0.72 | 0.60 | −0.92 | −0.29 | 1.21 |
| 3-Methylbutanal | −0.72 | −0.23 | 0.95 | −0.28 | 1.21 | −0.93 | −0.71 | −0.55 | 1.26 |
| 3-Methylbutanol | 0.14 | 1.07 | −1.2 | −0.93 | 1.11 | −0.18 | −0.66 | 0.81 | −0.15 |
| 3-Methylbutanoic acid | −0.66 | 1.28 | −0.62 | −1.27 | 0.50 | 0.77 | −1.26 | 0.51 | 0.75 |
| Ethyl 3-methylbutanoate | −1.24 | 0.94 | 0.31 | −1.27 | 0.29 | 0.97 | −0.81 | −0.49 | 1.30 |
| 3-Methylbutylacetate | 0.00 | 0.00 | 0.00 | 0.70 | 0.62 | −1.32 | 0.00 | 0.00 | 0.00 |
| ***Conditions-dependent*** | | | | | | | | | |
| Leucine | −0.40 | −0.56 | 2.56 | 0.56 | −0.52 | −0.42 | −0.77 | −0.22 | −0.24 |
| Ketoisocaproate | −0.47 | −0.08 | 2.27 | −1.05 | −0.63 | −0.65 | −0.4 | 0.01 | 1.00 |
| 3-Methylbutanal | −0.20 | 0.00 | 0.48 | −0.97 | −0.29 | −1.26 | −0.1 | 0.09 | 2.26 |
| 3-Methylbutanol | 0.52 | 1.72 | −1.21 | −1.29 | 0.04 | −0.81 | −0.05 | 0.83 | 0.25 |
| 3-Methylbutanoic acid | −0.48 | 1.02 | −0.45 | −0.86 | 1.29 | 1.61 | −1.11 | −0.55 | −0.48 |
| Ethyl 3-methylbutanoate | 0.39 | 1.57 | 1.23 | −1.03 | −0.73 | −0.6 | −1.03 | −0.73 | 0.94 |
| 3-Methylbutylacetate | −0.52 | −0.52 | −0.52 | 1.88 | 1.78 | −0.52 | −0.52 | −0.52 | −0.52 |

(BC: control; BP: alkaline pH; BS: high salt concentration).
